# Feasibility Study of Constructing a Screening Tool for Adolescent Diabetes Detection Applying Machine Learning Methods

**DOI:** 10.3390/s22166155

**Published:** 2022-08-17

**Authors:** Hansel Hu, Tin Lai, Farnaz Farid

**Affiliations:** 1 Atlas Advisors, Australia Pty Ltd., Sydney, NSW 2000, Australia; 2 School of Computer Science, Faculty of Engineering, The University of Sydney, Camperdown, NSW 2006, Australia; 3 Cybersecurity and Behavioural Science, School of Social Sciences, Western Sydney University, Penrith, NSW 2751, Australia

**Keywords:** diabetes detection, medical machine learning, adolescent diabetes prediction

## Abstract

Prediabetes and diabetes are becoming alarmingly prevalent among adolescents over the past decade. However, an effective screening tool that can assess diabetes risks smoothly is still in its infancy. In order to contribute to such significant gaps, this research proposes a machine learning-based predictive model to detect adolescent diabetes. The model applies supervised machine learning and a novel feature selection method to the National Health and Nutritional Examination Survey datasets after an exhaustive search to select reliable and accurate data. The best model achieved an area under the curve (AUC) score of 71%. This research proves that a screening tool based on supervised machine learning models can assist in the automated detection of youth diabetes. It also identifies some critical predictors to such detection using Lasso Regression, Random Forest Importance and Gradient Boosted Tree Importance feature selection methods. The most contributing features to Youth diabetes detection are physical characteristics (e.g., waist, leg length, gender), dietary information (e.g., water, protein, sodium) and demographics. These predictors can be further utilised in other areas of medical research, such as electronic medical history.

## 1. Introduction

Diabetes Mellitus (DM) is a chronic condition in which the amount of sugar in the blood is elevated [1]. The extra blood sugar could damage a wide range of the body’s organs, leading to heart attack, stroke, and problems with the kidneys, eyes, gums, feet, and nerves [1]. The most common type of DM is type 2 diabetes, accounting for approximately 90% to 95% of all diagnosed cases of DM [2]. On the other hand, the Centers for Disease Control and Prevention [3] has confirmed that Prediabetes Mellitus (PreDM) is a high-risk state for type 2 diabetes development where 5 to 10% of people with PreDM will progress to DM annually [4]. Alarmingly, both conditions are becoming prevalent among those younger than 20 years old due to the increase in childhood obesity [5]. For example, it is estimated by Imperatore et al. [6] that the prevalence of type 2 diabetes in young people in America is more than likely to quadruple between 2010 and 2050. In addition, it has been proven that DM in adolescent patients is far more difficult to treat when compared with adult patients [7]. Fortunately, numerous research and trials have proven that lifestyle and drug-based interventions could significantly lower the risk of diabetes development for those individuals with PreDM [8,9,10]. Therefore, it is critical for adolescents to have an accurate and intelligible detection tool to identify the PreDM/DM risks, such as the self-assessment screen tools published by the American Diabetes Association and the Centers for Disease Control and Prevention. Unfortunately, most of the screening tools are designed specifically for adults. For example, research by Vangeepuram et al. [11] has proved that those existing diabetes detection tools and guidelines did not adequately identify PreDM/DM status for adolescents. Therefore, this study aims to use the National Health and Nutrition Examination Survey (NHANES) datasets [12] together with machine learning models to accurately predict the risk of PreDM/DM and identify the critical information within the NHANES datasets that contribute to the PreDM/DM detection. The contributed model could be further developed into a simple screening tool. The following section explores the related research and studies to identify the problem and provide solutions to solve the problem. Section 3 explains the detailed process of utilising machine learning models to construct the screening tool, while the last two sections discuss the results and provide conclusions on the findings.

## 2. Background Study

This study focuses on peer-reviewed articles from January 2001 to August 2021. There are three main areas of interest: (i) the gap between the need for adolescent screening tools and the current approaches; (ii) studies that involve the application of machine learning models to diabetes detection; (iii) the development of interpretable machine learning models to identify the crucial predictors of diabetes detection.

On the one hand, various research and studies have established that a specially-designed and simple-to-use screening tool for adolescents to identify their risks of PreDM/DM is lacking. For example, Lobstein and Leach [13] have discussed the under-diagnosis of PreDM/DM among adolescents in the UK due to the lack of an effective screening tool. The current tools have mainly used the guidelines for adults to predict the PreDM/DM risks for adolescents. On the other hand, various research and studies have proved that machine learning models perform well in detecting PreDM/DM risk.

Vangeepuram et al. [11] used NHANES dataset to examine the performance of a published pediatric clinical screening guideline. The study is one of the first examinations of the recommended clinical procedure by health officials, which reveals a crucial shortcoming in the low accuracy of the existing screener for youth diabetes risk. In particular, the performance of the clinical procedure is compared against several machine learning-based classifiers derived from the NHANES dataset. The study [11] demonstrated that machine learning models performed better than screening guidelines in detecting youth diabetes and revealed the potential of using ML methods in clinical and behavioural health data.

Moreover, Yu et al. [14] achieved good performance in diabetes detection for adults with the Support Vector Machine (SVM) model and successfully identified the significant predictors for successful PreDM/DM detection among an extensive collection of independent variables. Similarly, Dinh et al. [15] applied the same process as the research of Yu et al. with more sophisticated machine learning models to boost the detection performance for adult subjects while discovering the critical predictors for PreDM/DM within the dataset. Therefore, this study applies the machine learning models of those prior research as a baseline to improve the performance and identify the predictors.

Unfortunately, most of the established research focused solely on adult subjects when collecting data for machine learning models, which partially explains why most screening tools obtain abysmal results when applied to detecting PreDM/DM in adolescents. For instance, in the research of Dinh et al. [15], which utilised machine learning models on the NHANES datasets for diabetes detection, all data collected from participants younger than 20 years old are discarded. Moreover, although some research applied machine learning models to PreDM/DM detection for adolescents, the results were not ideal. Although a similar approach from Dinh et al.’s research was taken by applying supervised machine learning models on the NHANES datasets, it did not conduct any feature selection method. Instead, they used five features from the screening tool published by the American Diabetes Association (ADA) and endorsed by the American Academy of Pediatrics (AAP) [16], namely BMI, family history of diabetes, race, hypertension, and cholesterol level. Unsurprisingly, the research results showed an unsatisfactory performance, with the best performing model yielding an F-score of 0.41, compared with the F-score of 0.68 from Dinh et al.’s research.

Therefore, this study aims to extract the established methods from adult diabetes research and apply them to adolescent diabetes detection to generate a model with adequate prediction capacity. However, a natural challenge exists after the prediction model, which is interpreting the predictors contributing to the detection. Johansson et al. [17] have suggested that although more sophisticated machine learning models usually achieve better performance, they cannot be easily understood by humans when compared with more straightforward machine learning such as simple linear models and tree-based models. Nevertheless, some research focuses on interpreting those more sophisticated models, and a method is highly related to this study. It is the feature importance method [18], which permutes the values of a variable, monitors the corresponding prediction accuracy to determine which variables have significant impacts on the prediction accuracy, and generates a list of essential features.

## 3. Screening Tool Design Details

**Figure 1:**
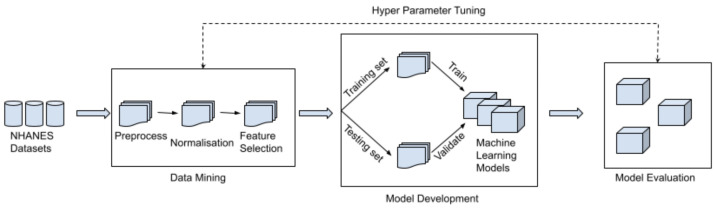
Workflow of the research.

Our research addresses the lack of a screening tool for youth diabetes but makes three adjustments to improve the detection performance of the models. Firstly, this research applies an exhaustive search to collect as many features as possible instead of only collecting features given by existing screening tools or guidelines. Secondly, feature selection methods select the features deemed essential for diabetes detection. Thirdly, instead of using the machine learning models from Vangeepuram et al.’s research [11], this study examined existing research in diabetes detection and included five fundamentally different machine learning models that are widely used, namely, Logistic Regression, Support Vector Machine, Random Forest, Extreme Gradient Boosted Tree and Weighted Voting Classifier. Figure 1 illustrates the workflow from original raw datasets through 3 different stages to the final detection of youth diabetes. The three stages are data mining, model development and model evaluation.

The scope of the study is limited to individuals between 12 and 20 years old with reliable and sufficient laboratory test results together with easy-to-obtain personal information, which does not require excessive examination or special medical devices or testing. The easy-to-obtain personal information not only sets fewer restrictions on data but, more importantly, makes the machine learning models less dependent on the data that is not easy to obtain. The laboratory test results include measurements of three biomarkers that are used to identify PreDM/DM status clinically [19,20,21]. They are plasma glucose level after an overnight fast (FPG), plasma glucose level two hours after an oral glucose load (2hrPG), and haemoglobin A1c (HbA1c), respectively. Therefore, any measurements requiring sophisticated testing or examination, such as the cholesterol level, are not included as they would not be suitable for building up the screening tool. The NHANES datasets are used for this study based on the aforementioned requirements. It is a program designed to systematically gather the health and nutritional conditions of the USA population. It is conducted and regulated by the National Center for Health Statistics (NCHS). The NHANES datasets combine various measurements and information of the participants from a wide range of data collection processes such as surveys, interviews, physical examinations and laboratory tests.

### 3.1. Data Preprocessing

We first begin by extracting the raw data from the NHANES database into a data frame, with as many features as possible. Firstly, the class label is assigned to each subject based on the three biomarkers mentioned above: (i) FPG, (ii) 2hrPG and (iii) HbA1c. The subjects are labelled as diabetic (label = 1) if either one of the three biomarkers is over the threshold. The three biomarkers are available from the NHANES laboratory test result dataset from 2005 to 2016. The remaining years in the collection lack the HbA1c test results and are excluded from this study. Otherwise, they are labelled as non-diabetic (label = 0). The labelling process is summarised in Table 1.

Secondly, all available information about the subjects is extracted to define the preliminary features of the dataset, with each feature representing a unique piece of information. Previous research to predict PreDM/DM using machine learning models and the NHANES datasets had provided an insight into the preprocessing and selection of the raw data [14,15,22], where all available information was extracted from the raw datasets to make sure there is no loss of data. Moreover, the study also examines the availability and continuity of all the features extracted to keep the ones that satisfy continuity and consistency conditions. NHANES contains survey data collected from different years. There are some inconsistent naming conventions, where some of the same variables used different feature names across different years. Moreover, some features were only collected in specific years, making a large number of missing values in many features. This might be due to the survey data from 1999 to 2022 having deviations in the data collection process.

We perform data cleaning by first recording features that contain the same semantic meaning into the same category. Then, features with over 50% of missing values are removed from the dataset. Normalisation was then applied to the numerical features. At the same time, the categorical features were transformed with one-hot encoding, where a new column is cleared for each categorical value with binary (0, 1) values to indicate whether the subject fits into each category. After performing the procedures mentioned above, the sample size of the final result is as follows. The consolidated dataset has been reduced to 2569, with 88 features between 2005 and 2016. It is also worth noting that the dataset is not balanced, as 1819 subjects are labelled as non-diabetic while the remaining 750 are labelled as diabetic.

### 3.2. Feature Selection

As this study aims to create a reliable model with a limited set of easy-to-obtain features, the feature dependence of the models is examined with various feature selection techniques to select the most essential and useful features out of the available 88 features. More importantly, feature selection can reduce the number of features without losing essential information and improve the efficiency and accuracy of the machine learning models. Firstly, highly correlated features are detected and displayed in pairs. When used in conjunction with other feature selection methods, this method could help identify the vital feature between the correlated pair, helping reduce the feature size by removing the other less critical feature.

Secondly, three different feature selection methods, namely Lasso Regression, Random Forest Importance and Gradient Boosted Tree Importance, are used to rank the importance of the features. They are the embedded feature selection methods that take advantage of the machine learning models used in the model development stage. Because this stage utilises the machine learning models that will be used for prediction, it guarantees to find features deemed necessary by the relevant models, improving the models’ detection performance.

**Figure 2:**
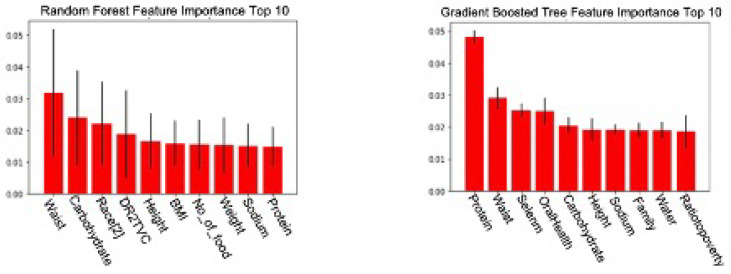
Top 10 important features of the (**left**) Random Forest Feature Importance Method and the (**right**) Gradient Boosted Tree Feature Importance Method.

Subsets of the Random Forest Importance model and Gradient Boosted Tree Importance model are displayed in Figure 2. Each model’s top 20 essential features are compared and cross-referenced to identify the frequently mentioned ones. The comparison highlights essential features that both models frequently choose, such as waist and height. We select those repeatedly appearing features suggested by different feature selection methods for the final pre-processed dataset, for example, age, gender, race, waist and height. Secondly, we examine the features that appear in at least one feature selection method by comparing them with existing research to determine their usability. For example, only the Gradient Boosted Tree Importance method marks oral health essential. However, current research has discovered a strong correlation between DM and deteriorated oral health [23,24], making oral health an eligible feature to be included in the final dataset.

After careful review, we select 18 features out of 88 for the model. These are age, gender, race, family history, body mass index (BMI), waist, height, weight, daily carbohydrate intake, daily sodium intake, daily protein intake, daily fat intake, daily sugar intake, daily dietary fibre intake, daily water intake, household income level, Vitamin B6 intake and oral health, respectively. Vangeepuram et al.’s research [11] used only five features from the APA screening tool: BMI, family history of diabetes, race, hypertension, and cholesterol level. On the other hand, the feature selection methods in our research have focused on a vastly different set of features, which partially explained the improved prediction performance of our model as compared to Vangeepuram et al.’s models. Our novel feature selection method contributes as follows:

- It ensures a more comprehensive examination of the data.
- It also assists in identifying the primary predictors of youth diabetes detection by removing some insignificant features. This constitutes an essential part of developing the screening tool.

### 3.3. Machine Learning Models

**Figure 3:**
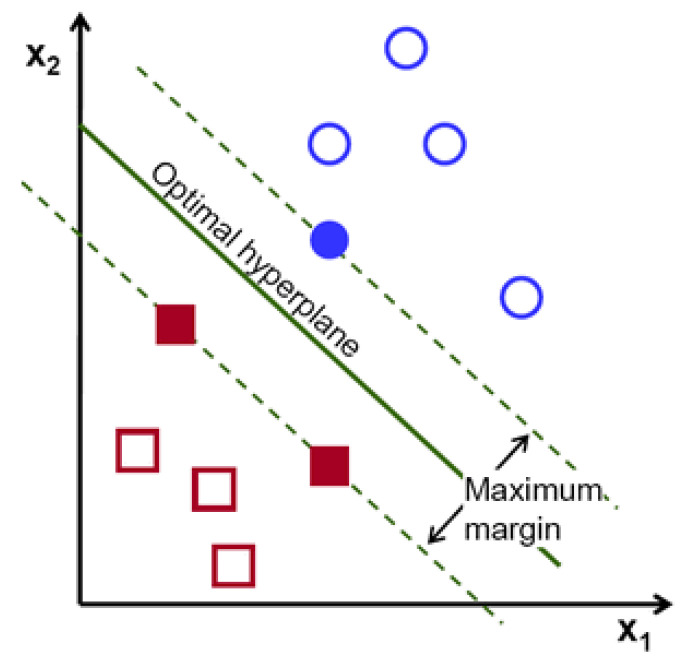
Support Vector Machine.

**Figure 4:**
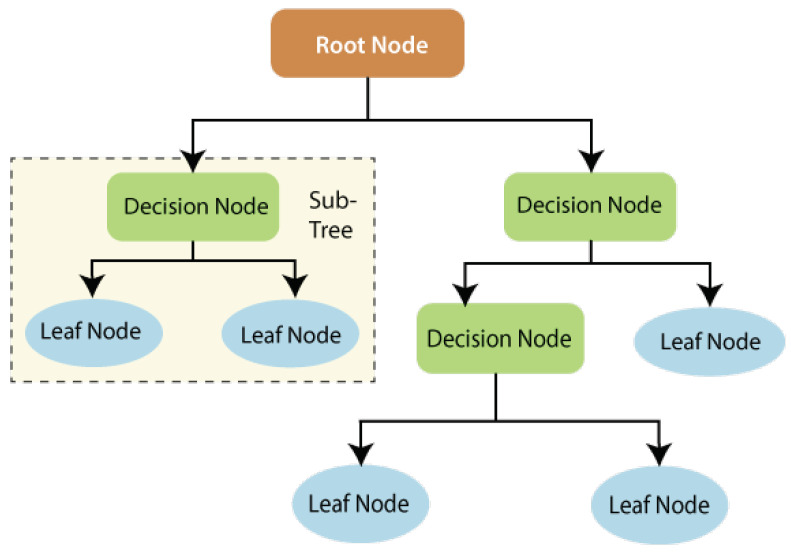
Decision Tree.

**Figure 5:**
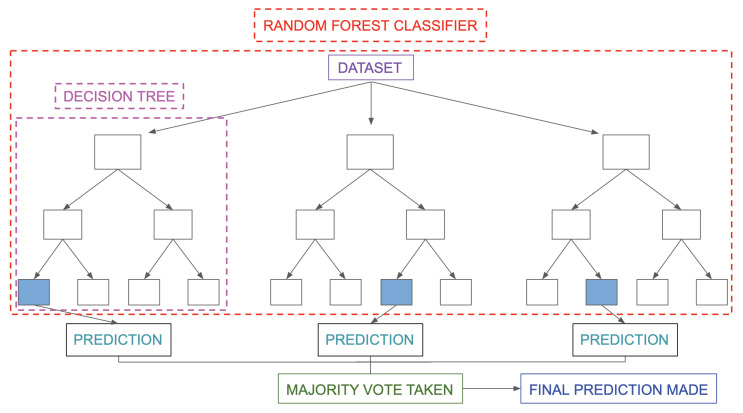
Random Forest.

**Figure 6:**
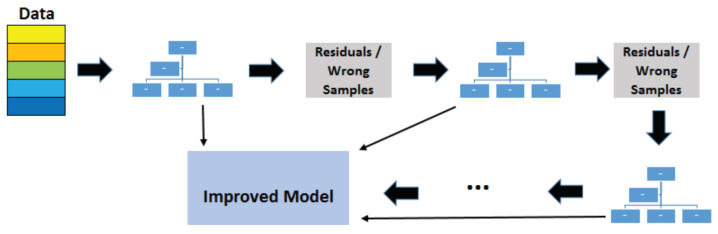
Extreme Gradient Boosted Tree.

In this study, various supervised machine learning models are used to detect the risk of PreDM/DM for adolescents. Supervised machine learning means the algorithm relies on data consisting of observed features and corresponding labels to build a model. After the model is built, it can predict a label when given a new set of features. In this case, the selected records from the NHANES datasets are the features, and whether the subjects have PreDM/DM inferred by the three biomarkers mentioned above is the label. All the machine learning models used in this study are briefly introduced below:

- Logistic Regression is a supervised machine learning model that is based on the probabilistic concept. Generally, it is used for classification problems. It generates probabilistic values between 0 and 1. In order to achieve this, the logistic function is applied to the simple linear regression as follows:
  (1) $$p=\frac{1}{1+e^{-(\beta_{0}+\beta_{1}X_{1}+\beta_{2}X_{2}+\cdots +\beta_{n}X_{n})\prime }}$$
  where *X* denotes the features, *p* denotes the probability of the detection, and β denotes the coefficients for each optimising features.
- The Support Vector Machine (SVM) is a supervised machine learning model applied to solve classification and regression problems. As illustrated by Figure 3, it provides prediction by proposing a boundary to separate the labels. It aims to achieve the complete boundary separation between different labels. To be specific, each subject in the dataset is plotted in an n-dimensional space, where n is the number of features of the dataset. The next step is to find the boundary known as the hyperplane to separate the data points in the space. However, it is not guaranteed that there is a boundary within the n-dimensional space. Kernel trick is used to transform the data into a higher dimension, where a boundary exists. SVM often yields better results than logistic regression but takes longer to develop due to the computational complexity in finding the boundaries.
- The Random Forest is a type of ensemble model that synthesises a collection of decision trees to achieve better performances in decision-making problems. As shown in Figure 4, it consists of three components: the root node, decision node and leaf node. The root node acts as a starting point. It includes all the features of the dataset. These are decision nodes representing a specific feature that divides the dataset into different sub-groups and leaf nodes that depict the label. Each decision tree keeps dividing the dataset into subgroups, which are further divided into other subgroups. This process continues until all the subjects within the subgroup share the same label. Each decision tree can be viewed as an analysis diagram with different outcomes. Finally, the predictions of all the trees within the collection are averaged to yield a final result, which can be summarised in Figure 5.
- The Extreme Gradient Boosted Tree (XGB Tree) is another ensemble model based on decision trees. Instead of utilising a collection of different decision trees, this model sequentially improves the decision trees based on the mistakes of the previous tree; the process is illustrated in Figure 6. The final result is weighted across the majority vote of all the trees.
- The Weighted Voting Classifier (WVC) combines the models above to generate results. The principle of this method is to use a weighted ensemble method to take advantage of the strengths of all the models. Specifically, it takes multiple predictions of separate models and averages them with weights based on model performances.

Logistic Regression and SVM are comparatively simple models compared with all the other models. They have been extensively used and applied in the disease detection area. They are used as baseline models in many research studies. Yu et al.’s [14], Dinh et al’s [15], and Vangeepuram et al.’s research [11] have included Logistic Regression and SVM models. In addition, Random Forest and Extreme Gradient Boosted Tree are applied to explore the non-linear relationships that the Logistic Regression and SVM cannot discover. Moreover, Random Forest and Extreme Gradient Boosted Tree are tree-based models, making it easier to interpret the important features for successful detection. Finally, as mentioned earlier, the WVC is included to explore the potential benefit from the strengths of all the models.

### 3.4. Model Development

After the data preprocessing stage, we split the consolidated dataset into training and testing sets for developing and evaluating machine learning models. The training set is used in the training phase, where models are constructed. During the training phase, a grid-search method with 10-fold stratified cross-validation is applied to generate the best model parameters, which are then utilised to compose the best model. On the other hand, the testing set generates a set of quantitative metrics measuring the model performance. It successfully compares and evaluates all the models.

### 3.5. Evaluation Metrics

In this section, the quantitative metrics for the model performance are introduced. The building blocks for the metrics can be summarised into four different concepts, True Positive (TP), True Negative (TN), False Positive (FP) and False Negative (FN). For this study, a TP is when the model correctly predicts a diabetic label for a subject that has a diabetic label. Similarly, a TN is the model’s correct prediction of a non-diabetic label. On the other hand, an FP is when the model predicts the diabetic label, but the actual label is non-diabetic. An FN can be defined using the same principle. The confusion matrix to illustrate the four concepts is listed in Table 2.

We consider four metrics to measure the model performance based on the above concepts. Firstly, precision ($\frac{TP}{TP+FP}$) is used to measure the proportion of correctly identified diabetic subjects out of all the subjects being detected as diabetic by the models. The second metric is Recall ($\frac{TP}{TP+FN}$), which measures the proportion of all the diabetic subjects that are identified correctly by the models out of all the true diabetic subjects of the test set. Thirdly, the F1 score ($2\times \frac{precision\times recall}{precision+recall}$) is used as a harmonic average of precision and recall to assess the prediction of the models for true diabetic subjects in conjunction with the false positives. Finally, the area under the curve (AUC) and receive operating characteristic (ROC) are used to explore further the relationship between precision and recall of each model. ROC is a probability curve that plots recall against a false positive rate ($\frac{FP}{TN+FP}$) while AUC summarises the ROC curves and measures the ability of the model to distinguish between labels. All four metrics have values ranging from 0 to 1, with a higher score indicating better performance.

### 3.6. Statistical Testing

Apart from the evaluation metrics mentioned above, it is also essential to consider whether the model performs better than the other by chance. Therefore, the 5x2cv paired *t*-test proposed by Dietterich [25] has been utilised to determine if the differences between different models are significant. The test procedure is to split the data evenly into a training set and a testing set five times. For each split, two target models are trained with the training set and evaluated with the test set. In addition, the training set and testing set are rotated and are used to generate the second evaluation performance, which could generate two performance difference measures:

(2) $$p^{\left(1\right)}=p_{A}^{\left(1\right)}-p_{B}^{\left(1\right)}$$

(3) $$p^{\left(2\right)}=p_{A}^{\left(2\right)}-p_{B}^{\left(2\right)}$$

The mean and variance of the differences can be estimated as:

(4) $$\bar{p}=\frac{p^{\left(1\right)}-p^{\left(2\right)}}{2}$$

(5) $$s^{2}={\left(p^{\left(1\right)}-\bar{p}\right)}^{2}+{\left(p^{\left(2\right)}-\bar{p}\right)}^{2}$$

The variance is calculated for the five splits and then used to calculate the t statistic as follows:

(6) $$t=\frac{p_{1}^{\left(1\right)}}{\sqrt{\left(1/5\right)\sum_{i=1}^{5}}s_{i}^{2}}$$

### 3.7. Model Interpretation

As previously introduced in Section 2, we use the feature importance method to interpret the models. This method’s principle is to permute a feature’s value to monitor the change of prediction error. An important feature will significantly increase the model error after permutation and vice versa for unimportant features. In this study, after the best model is constructed for each machine learning model, the testing set is fed into the best model with the feature importance process. It generates a list of important features for the models.

## 4. Results

**Figure 7:**
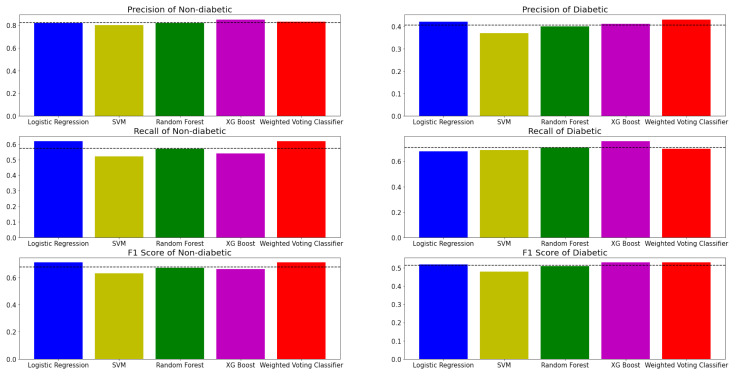
Performance of machine learning models in Diabete detection with Non-diabetic and Diabetic class labels, in terms of precision, recall and F1 score.

Figure 7 shows the evaluation metrics of five models in classifying PreDM/DM risks with the selected features and class labels inferred from three biomarker criteria. Evidently, all the models have more accurate predictions for the non-diabetic label. There could be several possible factors that induce performance differences across labels. Firstly, the predictability of diabetes within the population has always been challenging across the population, even with recommended screening tools [26]. Moreover, the dataset is imbalanced where 70% of the subjects have non-diabetic labels. For example, in an extreme case where a model predicts all subjects to be non-diabetic, the evaluation metrics of the non-diabetic label could still appear satisfactory due to the domination of the non-diabetic label. Therefore, the evaluation metrics of the diabetic label are also essential. Based on Figure 7 and Table 3, the performance of the models in detecting diabetic labels shows significant improvement when compared with previous research conducted by Vangeepuram et al. [11] using the same NHANES datasets. According to Table 3, it is comparatively easy to observe that even the worst performing model, SVM, generates a dominating performance over the best model in Vangeepuram et al.’s research. This could be caused by the fact that the previous research had only included five features, which could lead to a significant loss of information compared to the exhaustive feature search in this study. The choice of features matters [27], and in general, more features allow the ML model to model a more flexible predictive model [28]. Table 4 is constructed to illustrate the results. For readability reasons, the numeric value of the *t*-test results is replaced with a check-mark when model A’s performance is better than model B’s. In addition, another characteristic of the table is that the performance of model A is always better than that of model B. The *t*-tests are performed using a *p*-value of 0.05. Based on Table 3 and Table 4, it is clear that the difference in performance between more sophisticated (MVC, XGB Tree and Random Forest) models and simpler models (Logistic Regression and SVM) were not always significant. One possible explanation is that the model may perform better than the other by chance. For example, XGB Tree showed mixed results, with only a difference in performance against SVM being statistically significant. On the other hand, Random Forest showed a more stable result with a significant performance difference against Logistic Regression and SVM. In the case of MVC, the difference in performance against SVM and Random Forest was statistically significant.

**Figure 8:**
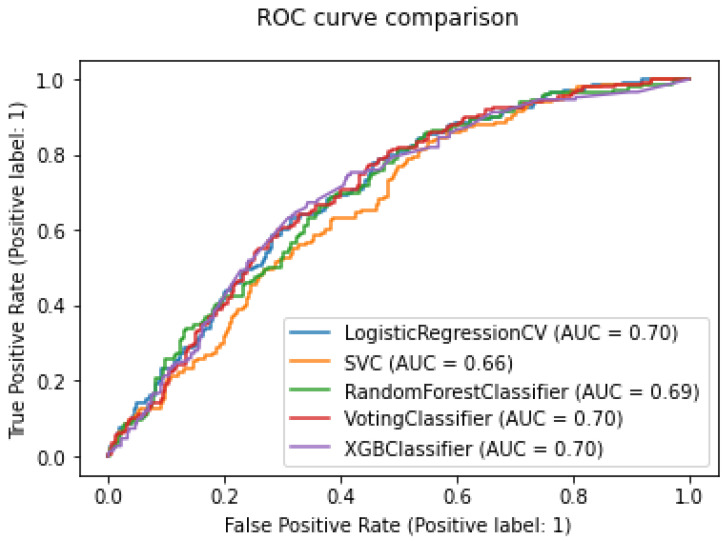
ROC curves of different models.

Figure 8 displays the models’ ROC curves. When compared in conjunction with Table 3 and Table 4, it is immediate that more complicated models will yield a more performant result. For example, the Random Forest model and XGB Tree evaluation metrics are comparatively higher than those of Logistic Regression and SVM. More importantly, it is evident that the WVC achieved the best overall performance, with the AUC score being the highest at 0.71.

**Figure 9:**
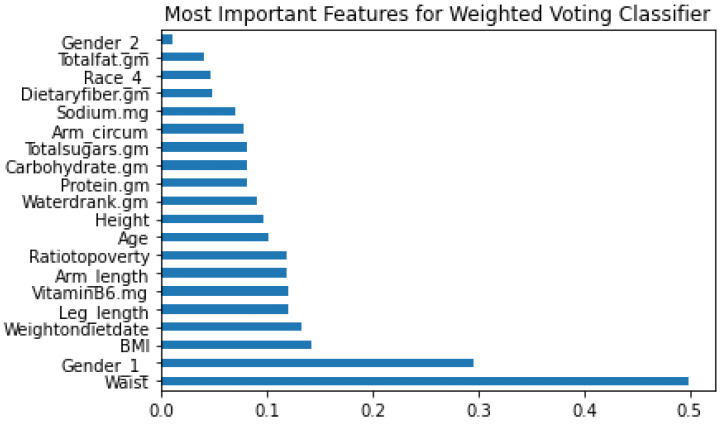
Most Important Features for Weighted Voting Classifier.

Figure 9 shows the importance of 20 features contributing to the PreDM/DM detection in the WVC. Figure 4 shows that features such as waist, gender, BMI and leg length are essential factors for detecting DM/PreDM. Moreover, waist size and gender (male) are significant in determining the PreDM/DM risks, with their importance scores being drastically higher than others.

## 5. Discussion

This study conducts an exhaustive search on the NHANES datasets to develop five different machine learning models for comparative analysis based on the model performances in detecting PreDM/DM. Compared to the approach by Vangeepuram et al. [11], all the models in this study display better performance. An essential contribution of this study is to identify the critical features that substantially contribute to the detection of PreDM/DM. For example, the best model, WVC, can discover features with physical characteristics (waist, leg length, gender), dietary information (water, protein, sodium) and demographics (race). The research showcased a significant way of achieving promising interpretable results in detecting PreDM/PDM, which paves the way to understanding the essential features for such successful detection. Further adoption of the models into a real-world application can be a combination of a paper and web-based screening tool, where a questionnaire can be used to collect information regarding those identified features and assess participants’ PreDM/DM risk. This screening tool can close the gap between the urgent need and the lack of such a tool.

## 6. Conclusions and Future Work

This research developed an explainable machine learning-based model for predicting youth diabetes. A series of Supervised machine learning methods such as Logistic Regression, SVM, Random Forest, XGB Tree and WVC are applied. The best model, WVC, determines a significant number of features that can efficiently detect PreDM/DM in adolescents. It also achieves a promising result with an AUC score of 71%.

However, despite the improvement and promising findings, this study still has a few limitations. Firstly, the sample size is relatively small with 2569 subjects, especially when compared to similar research conducted with adult subjects, for example, the research of Dinh et al. [15] using the same NHANES datasets on PreDM/DM detection for adults has over 15,000 subjects. Although this would not substantially affect the results, a larger sample size is always favourable. Secondly, there is no differentiation between type 1 and type 2 diabetes in the NHANES dataset, and this study takes the same approach to detect the whole diabetes group. Furthermore, some information, such as physical activity data from monitor devices, in the NHANES dataset, is not released to the public due to confidentiality and censorship. Including the extra information could potentially disclose more critical factors that contribute to preventing PreDM/DM. Future work of this study includes collecting more subjects and features from the NHANES datasets to explore the findings using existing models, which could mitigate the limitations mentioned above. Including more completed feature sets in ML models will allow the health officials to focus on collecting the most critical features for diabetic detection. Moreover, more advanced machine learning models, such as neural networks, could boost performance, requiring more sophisticated model interpretation methods to find the critical factors.

## Figures and Tables

**Table 1:** The Clinical guideline used to define Diabetes/Non-diabetes (preDM/DM) status.

Criteria	Classification
plasma glucose level after an overnight fast (FPG) ≥ 100 mg/dL	Diabetes/1
plasma glucose level two hours after an oral glucose load (2hrPG) ≥ 140 mg/dL	Diabetes/1
hemoglobin A1c (HbA1C) ≥ 5.7%	Diabetes/1
None above	Non-diabetes/0

**Table 2:** Confusion Matrix.

True Label	Model Prediction	
	Non-Diabetic	Diabetic
Non-Diabetic	True Negative	False Positive
Diabetic	False Negative	True Positive

**Table 3:** Evaluation metrics of the models from previous research and this research.

	AUC	Precision	Recall	F1 Score	Accuracy
Best model from previous research	N/A	0.35	0.36	0.35	N/A
Logistic Regression	0.70	0.42	0.68	0.52	0.64
Support Vector Machine	0.66	0.37	0.69	0.48	0.57
Random Forest	0.69	0.40	0.71	0.51	0.61
Extreme Gradient Boosted Tree	0.70	0.41	0.76	0.53	0.61
Weighted Voting Classifier	0.71	0.43	0.70	0.53	0.64

**Table 4:** 5x2cv paired *t*-test.

Classifier A	Classifier B	Result
Weighted Voting Classifier	Logistic Regression	
	SVM	🗸
	Random Forest	🗸
	Extreme Gradient Boosted Tree	
Extreme Gradient Boosted Tree	Logistic Regression	
	SVM	🗸
	Random Forest	
Random Forest	Logistic Regression	🗸
	SVM	🗸
SVM	Logistic Regress	

## Data Availability

The datasets analysed during the current study are available in the National Center for Health Statistics repository, accessed 1 March 2022, https://wwwn.cdc.gov/Nchs/Nhanes/.
